# Maternal recognition of pregnancy in the mare: does it exist and why do we care?

**DOI:** 10.1530/REP-20-0437

**Published:** 2021-04-09

**Authors:** Aleona Swegen

**Affiliations:** 1 Nuffield Department of Women’s and Reproductive Health, University of Oxford, Oxford, UK; 2 Priority Research Centre for Reproductive Science, University of Newcastle, Callaghan, New South Wales, Australia

## Abstract

Maternal recognition of pregnancy (MRP) is a process by which an early conceptus signals its presence to the maternal system and prevents the lysis of the corpus luteum, thus ensuring a maternal milieu supportive of pregnancy continuation. It is a fundamental aspect of reproductive biology, yet in the horse, the mechanism underlying MRP remains unknown. This review seeks to address some of the controversies surrounding the evidence and theories of MRP in the equine species, such as the idea that the horse does not conform to the MRP paradigm established in other species or that equine MRP involves a mechanical, rather than chemical, signal. The review examines the challenges of studying this particularly clandestine phenomenon along with the new tools in scientific research that will drive this quest forward in coming years, and discusses the value of knowledge gleaned along this path in the context of clinical applications for improving breeding outcomes in the horse industry.

## Introduction

It is remarkable that horses have been domesticated, bred and studied by humans for thousands of years, yet we have struggled to define one of the most fundamental elements of their reproductive function – maternal recognition of pregnancy. Maternal recognition of pregnancy (MRP) is a term first coined by Roger Short (Short 1969). At this time, the universal role of the corpus luteum (CL) in maintaining a pregnant state in mammals had been established, but the idea that a conceptus, at its very early stages of development, was capable of actively influencing the maternal organism and the fate of the CL was only nascent. Evidence for a functional relationship between the embryo and the corpus luteum dates to the mid-1960s in sheep (Moor & Rowson 1966) and the end of the 70s in cattle (Betteridge *et al.* 1980, Northey & French 1980), all from studies aimed primarily at improving embryo transfer. The emergence of MRP as a defined concept in reproductive biology served as an early acknowledgement of this embryo–maternal communication and now refers to the process by which the conceptus communicates its presence and ultimately results in prolonged survival, rather than lysis, of the CL, thus preventing a return to oestrus. In stark contrast to the widely conserved need for a CL in pregnancy, the MRP signalling molecules emitted by concepti of different species are striking in their diversity, ranging from single chain proteins to steroid hormones (Bazer 2015). Discovery of the human MRP signal, human chorionic gonadotrophin (hCG), facilitated the development of a widely used pregnancy test and ushered in a new era in fertility intervention, based on luteal phase support for IVF pregnancies and those at risk of spontaneous abortion (Casper *et al.* 1983). Meanwhile, the equine MRP mechanism has remained elusive, despite decades of research and incremental advances in our understanding of the downstream effects of the purported signal. Several excellent reviews have summarised the progress made on uncovering this perplexing mechanism in the equine species (Klein 2015*a*,^2016^, Stout 2016)). In this piece, I seek to examine, rather, the approaches to, and implications of understanding MRP in the horse. Some have suggested that the horse, in all its uniqueness, does not conform to the paradigm of having a clear chemical signal, emitted by the embryo, that directly delays maternal luteolysis. Will a critical re-examination of experimental evidence support this notion? An alternative explanation is that current scientific methods and platforms have evolved to the point that a simplistic, linear relationship between an embryo-secreted product and an endocrine event is no longer relevant, as we start to see the ‘greys’ between the ‘black and white’ of earlier scientific discoveries. Hence, I shall discuss what the ‘omics era means for MRP research and how this research community can make the most of exciting new developments and experimental tools emerging in other fields. Oftentimes the quest for the equine MRP is framed as the key to preventing early pregnancy loss and developing a simple new pregnancy test. How realistic are such promises and what other clinical relevance might the mechanisms involved in MRP, if discovered, hold? In the following sections, I examine concepts, approaches and consequences of MRP in the horse, and argue for a need to reframe the investigation of equine pregnancy in order to make progress that is both clinically relevant and biologically meaningful.

## Looking back: a brief history of maternal recognition in the horse

The equine conceptus differs distinctly from those of other ungulates; the equine blastocyst does not elongate and instead remains spherical, becomes encased by a glycoprotein capsule that forms beneath the zona pellucida just before its shedding, and embarks on a rather vigorous migration throughout the uterus (reviewed in detail by (Stout 2016)). Remarkably, the embryo survives in the absence of vascular communication with its dam until around day 40 post-ovulation when definitive placental formation begins. Despite this, the majority of conceptuses successfully signal their presence and are capable of eliciting substantial physiological changes at multiple levels–—in the immediate (luminal) uterine environment, the maternal reproductive tract (endometrium and CL) and the systemic circulation. Yet, precisely how these changes integrate to establish the pregnant maternal state remains to be pieced together.

The mare’s CL persists for 14 days after ovulation irrespective of whether a blastocyst is present in the uterus, and after this period luteolysis takes place only if no embryo is present. Identifying the critical time window during which signalling must occur to prevent luteolysis has been the subject of numerous studies and varying approaches; a non-exhaustive chronological summary of these is presented in Table 1. As for the molecular interactions that constitute equine MRP, the precise timing of a defined ‘critical window’ for MRP remains ambiguous but it appears that various stages of the MRP cascade become active during the period between 10 and 16 days post-ovulation. Throughout the 1980s, the central role of the hormone prostaglandin F (PGF) in this timeline of luteolysis was recognised and defined, noting that the presence or absence of an embryo affected the production or secretion of PGF from the endometrium. Crucially, it was soon observed that despite lower PGF release from pregnant endometrium *in vivo*, the *in vitro* production capability of endometria from pregnant mares appeared to be the same as that of non-pregnant mares: endometrium from pregnant mares, as soon as it was removed from the immediate vicinity of the conceptus, would produce (at least) the same levels of PGF as non-pregnant endometrium (Vernon *et al.* 1981, Berglund *et al.* 1982). It was only when conceptus membranes were included in the incubation that the level of PGF detected in the endometrial culture medium was clearly reduced (Sharp *et al.* 1984). It thus became evident that the effect of the conceptus on endometrial PGF production is transient. This was further confirmed through multiple studies, both *in vivo* and *in vitro*, that clearly demonstrated the ability of the conceptus to invoke a direct suppression of prostaglandin F_2α_ (PGF_2α_) production in the endometrium, the suppression being attributed to a reduction of both the activity and expression of the cyclooxygenase-2 enzyme (COX-2; prostaglandin-endoperoxide synthase 2; PTGS2) (Boerboom *et al.* 2004, Ealy *et al.* 2010).

**Table 1:** A chronology of studies providing evidence on timing of maternal recognition of pregnancy in the horse.

Reference	Time examined (days post-ovulation)	Relevant outcome	Experimental approach	Results relevant to MRP	Implications for MRP timing
Leith & Ginther (1984)	Days 9 to 17	Mobility patterns of the embryonic vesicle	Ultrasonography of pregnant mares	On day 9, conceptus mobility was minimal. Mobility increased on day 10 and reached an apparent plateau from day 11 to 14.	Suggests MRP signalling active during days 10–14
Sharp *et al.* (1984)	Days 4 to 20	Endometrial and uterine PGF content; response of endometrium to presence of conceptus	PGF measured in uterine luminal flushings and endometrium;* in vitro* incubations of endometrium alone vs endometrium in the presence of conceptus membranes.	Uterine luminal PGF and endometrial PGF/*in vitro* production peaked on/around day 14 in cycling mares. Endometrial PGF content remained lower in pregnant mares until day 20, but* in vitro* production capability of endometria from pregnant mares continued to increase post day 14. Co-incubation of endometrial tissue and conceptus membranes* in vitro* reduced PGF production.	Embryos exert MRP by blocking PGF production; the process is engaged before day 14; day 14 endometrium is responsive to MRP.
Goff *et al.* (1987)	Days 9 to 14	Oxytocin responsiveness of endometrium	Pregnant and non-pregnant mares were given oxytocin intravenously and plasma PGF metabolites measured.	In non-pregnant mares, the increase in plasma PGFM response to oxytocin was greater at day 13 than day 11; no significant increase in response to oxytocin days 9–14 in pregnant animals.	Non-pregnant endometrium becomes responsive to oxytocin between day 11 and 13; MRP expected to be initiated before this time.
Sissener *et al.* (1996)	Conceptus days 9, 12, 13, and 16; uterine biopsies on day 14.	Age of the conceptus inducing maximal suppression of PGF_2α_	Embryos recovered and co-incubated with endometrium, PGF_2α_ measured in all samples.	Day 12 conceptuses significantly suppressed endometrial PGF_2α_ secretion compared with that of endometrial tissue incubated alone.	Embryo emits MRP signal at day 12 (other timepoints cannot be excluded).
Starbuck *et al.* (1998)	Days 10, 14, 18	Oxytocin responsiveness of endometrium; role of oxytocin receptors	Pregnant and non-pregnant mares injected with oxytocin; circulating concentrations of PGFM measured; concentrations of oxytocin receptor measured in endometrial biopsy samples.	PGFM concentrations were increased after oxytocin administration on days 10/14/18 in cyclic mares, but only on days 10/18 in pregnancy. Suppression of oxytocin receptors in the endometrium occurred between day 10 and 16.	Oxytocin responsiveness/receptor expression is blocked in pregnant mares after day 10 and before day 16, suggesting possible window of MRP.
Stout *et al.* (1999)	Days 8 to 20	Oxytocin responsiveness of endometrium	Oxytocin vs saline administered to mares via continuous infusion, monitored for luteolysis.	Continuous administration of oxytocin on days 8–20 abolished luteolysis in most mares. When oxytocin treatment began on day 10, instead of day 8, luteolysis occurred in two of five mares.	Oxytocin responsiveness begins on or about day 10.
Ababneh *et al.* (2000)	Day 13 embryos	Ability of embryos to suppress endometrial PGF_2α_	Embryos were collected from pregnant mares 13 +/—− 0.5 days after ovulation and cultured for 24 h; conceptus media assayed for antiluteolytic activity by measuring endometrial PGF_2α_ synthesis* in vitro*.	Antiluteolytic activity detected in embryo-conditioned culture medium at 12, 18 and 24 h.	Day 13 conceptuses are capable of emitting MRP signal.
Stout & Allen (2002)	Days 14–18 (cycling mares); days 12–32 (pregnant mares).	Effect of pregnancy on uterine luminal prostaglandin levels	Influence of pregnancy on concentrations of prostaglandins in the uterine lumen examined	PGF_2α_ concentrations reached high values in uterine flushings recovered from cyclic mares during days 14–16 after ovulation, but were negligible in flushings from pregnant mares at this time.	Conceptus attains MRP during days 12–16.
Boerboom *et al.* (2004)	Days 10, 13, and 15	Response of endometrium to presence of conceptus.	Prostaglandin synthesis enzymes examined in endometrial biopsies obtained from cycling mares on days 10, 13 and 15; pregnant mares on day 15.	COX-2 expression higher at day 15 in cycling endometrium relative to other timepoints. COX-2 expression in day 15 pregnant endometrium was similar to day 10 and day 13 cycling animals, suggesting that presence of the conceptus blocks the induction of COX-2.	MRP has been activated by day 15 via COX-2 suppression
Ealy *et al.* (2010)	Day 14	Ability of embryos to suppress endometrial PGHS2	Relative abundance of PGHS2 mRNA measured in endometrium derived from estrous cyclic and pregnant mares on day 14 post-ovulation, co-incubated with conceptus-conditioned medium.	Exposing endometrial explants to conceptus secretions decreased PGHS2 mRNA abundance.	Endometrium is responsive to PGF synthesis inhibition on day 14.
Wilsher *et al.* (2010)		Ability of mare to recognise pregnancy following asynchronous embryo transfer	Day 10 horse embryos were transferred non-surgically to recipient mares that had ovulated 9, 7, 6, 5, 4, 3, 2 or 1 day after, on the same day, or 2 or 4 days before the donor.	Luteostasis was achieved in recipient mares when day 10 embryos were transferred to recipient mares at any stage of asynchrony between −9 and +2 days with respect to the donor, i.e. there is a wide window for establishment of pregnancy following embryo transfer to asynchronous recipients.	The luteolytic cascade in the non-pregnant mare is not initiated before day 12 after ovulation.
Wilsher & Allen (2011)	Days 6, 8, 10, 12, 14	Endometrial responsiveness to PGF synthesis inhibition	Oestradiol and/or oils infused into the uterine lumen of cycling mares on days 6, 8, 10, 12 or 14 post-ovulation; other mares received intrauterine infusion of fractionated coconut oil, peanut oil, mineral oil or oestradiol in mineral oil on day 10 post-ovulation. Luteal function monitored by plasma progesterone assay.	Intrauterine administration of oestradiol in coconut oil delayed luteolysis at days 8, 10, 12, or 14 but was less effective at day 6. Coconut oil alone or peanut oil administered at day 10 induced the same high rate of luteal persistence. A possible interpretation is that fatty acid inhibition of phospholipase A2 limits the ability of endometrium to synthesise prostaglandins.	Endometrium appears responsive to PGF synthesis inhibition on days 8–14 but not on day 6.
Camozzato *et al.* 2019	Days 7, 10, 13	Histological changes in the endometrium of pregnant vs cycling mares	Endometrial biopsies from pregnant and non-pregnant mares assessed histologically; plasma progesterone concentrations measured daily.	In pregnant vs non-pregnant mares, differences in vasculature observed on days 7–13, differences in endometrial histology by day 7, differences in histotroph secretion by day 10. No difference in progesterone levels in pregnant vs cycling mares.	Some form of embryo-maternal dialogue is occurring from at least day 7.
Klohonatz *et al.* (2019)	Days 9 and 11	Ability of embryos to suppress endometrial PGF_2α_	Endometrial biopsies from pregnant and non-pregnant mares on days 9 and 11 co-incubated in contact with embryos of corresponding age; PGF measured in culture medium.	In day 11 samples from non-pregnant mares, presence of an embryo decreased PGF secretion compared to control samples from non-pregnant mares. On day 9, there was no change in PGF secretion in the presence of an embryo.	MRP occurring at day 11 but not yet detectable by this method at day 9.

COX-2, cyclooxygenase 2; PGF, prostaglandin F; PGFM, prostaglandin metabolite, 13,14-dihydro-15-ketoprostaglandin F2 alpha ; PGHS2, prostaglandin G/H synthase 2.

It was later suggested that spatial distribution of the purported signal was also necessary for luteal support, as demonstrated in a study where conceptus mobility was restricted to certain parts of the uterus using ligation (McDowell *et al.* 1988). This was a landmark discovery in equine reproduction and revealed one of the equine pregnancy’s most remarkable and unique features. To this day, it is not clear what exactly drives the biological requirement for conceptus mobility —– perhaps a mechanical interaction between the embryo and the endometrium, chemical instability of the MRP signal, or a need for the embryo to receive adequate nutrition through the secretions of the endometrial glands to maintain its survival. Some have questioned the necessity for mobility as Wilsher* et al* observed that in some mares receiving transferred embryos, embryo movement ceased at day 9, yet luteostasis still occurred (Wilsher *et al.* 2010). Nevertheless, most commonly it is asserted that the embryo needs to disseminate an MRP signal to the entire endometrial surface for the signal to be received and transmitted appropriately. While the findings of McDowell* et al.* that regard conceptus mobility area and progesterone levels support this hypothesis, they leave open the question of whether the MRP signal is a single chemical released directly by the conceptus, a mechanical interaction, or a series of events.

If physical features of the embryo are more important for MRP than the compounds it releases, then presumably embryo fragments or homogenates, with retained secretory activity, would fail to maintain luteal lifespan. Attempts have been made to explore this via studies employing ‘trophoblastic vesicles’ —– embryo fragments cultured to form multiple independent conceptus-like structures. Ball* et al.* pioneered this technique in the horse (Ball *et al.* 1989). Initial experiments tentatively suggested that these structures could prolong luteal lifespan, but further investigation indicated this may not be the case. Although the vesicles did secrete an array of polypeptides in a similar fashion to an intact conceptus, vesicles from days 12 to 14 conceptuses transferred to mares at day 10 or 12 post-ovulation did not prolong luteal maintenance (Ball *et al.* 1991), suggesting that the physical features of the conceptus—–that is, transit through the uterine lumen and constant contact with the endometrium—–might indeed be essential components of the MRP process.

Other studies have sought to decipher the chemical properties of the MRP signalling compound. Co-incubation of day 14 conceptus membranes with day 14 endometrial explants in combination with selective dialysis suggested that a conceptus factor between 1 and 6 kDa molecular mass was responsible for inhibiting endometrial prostaglandin production (Sharp *et al.* 1989). Culture medium, in which embryos flushed at day 13 had been incubated, inhibited endometrial PGF_2α_ synthesis; this *in vitro* antiluteolytic activity was maintained only by the 3–10 kDa fraction of medium and was unaltered by treatment with the enzyme proteinase K (Ababneh *et al.* 2000). The latter observation is noteworthy as virtually all proteins are susceptible to lysis by proteinase K—–its non-specific proteolytic action is what makes it a widely used tool for ‘cleaning up’ preparations of DNA and RNA, that is, for freeing them of protein contamination. The exceptions are prion proteins and proteinase K itself. Thus, Ababneh’s result implies that the MRP is more likely a non-protein entity (e.g. a miRNA), or a protein whose inhibition of PGF_2α_ synthesis stems directly from peptide-level activity and is thus unhindered by breakdown of the protein into its peptide components. The same study also showed that the anti-prostaglandin activity is abrogated by dextran-coated charcoal adsorption. This finding is somewhat less revealing about the chemical nature of MRP; charcoal adsorption is readily affected by variations in concentration of reagents, time, temperature, pH, and other experimental factors. Nevertheless, it does suggest that the active compound is more likely to be a free, small molecule than a large complex, in line with the results of the proteinase K and molecular size experiments.

Thus a sufficient accumulation of evidence for MRP in the horse does exist but much more remains to be discovered about its timing, mechanism and chemical nature. The ability to mimic the anti-prostaglandin response *in vitro*, efficient non-surgical lavage of blastocyst-stage embryos and the evolution of new, sensitive analytical platforms must now coalesce to fuel further experiments that build on those discussed above and thus uncover more details about the MRP mechanism in the horse.

## Searching for clues: mechanical vs chemical MRP

The subject of equine MRP has given rise to some heated discussions over the years and in many instances, the suggestion is put forward that it is the physical contact of the embryo alone that triggers MRP in the mare. This view probably arises in part from the long-standing practice of using glass marbles or other inert objects, placed in the uterine lumen, to prevent mares from cycling. The mechanism behind this phenomenon remains uncertain. Attempts to clarify this process have been largely inconclusive with mixed effects of intrauterine devices (IUDs) on prolongation of the luteal phase and some, but inconsistent, reduction in systemic prostaglandin metabolite levels (Rivera del Alamo *et al.* 2008). Furthermore, whether these effects are mediated through COX-2 enzyme reduction has not been adequately explored (Rivera del Alamo *et al.* 2018). Physical contact with the endometrium–at least such that can be mimicked by an inert IUD–seems to be insufficient to consistently delay luteolysis in all instances, and it remains to be determined whether IUD-induced luteal maintenance parallels the mechanism involved in physiological MRP or is coincidental (i.e. driven by a completely different mechanism of PGF reduction).

Mechanisms underlying the abovementioned ‘marble effect’ have proven challenging to examine. Looking to empirical evidence from other body systems and other species, there is certainly some potential for mechanoreceptors in the endometrium or myometrium to be involved in equine MRP. In bony tissue, where the role of mechanotransduction is much better established, interactions exist between mechanical stimuli and the expression of COX-2, whereby prostaglandin E2 synthesis is increased in response to mechanical forces via the formation of focal adhesions (Hughes-Fulford 2004). Thus, it is conceivable that the marble and/or the conceptus could trigger a mechanoreceptor response that modulates COX-2 activity and affects prostaglandin production in the endometrium.

Pursuing the trail of mechanical effects of the conceptus in the mare’s endometrium, Klohonatz* et al.* detected miRNAs in the circulation of pregnant and non-pregnant mares that appear to differentially target focal adhesion molecules (FAMs) at the time of the expected MRP (Klohonatz *et al.* 2016). The theme was further pursued in a subsequent study, looking more closely at the role of focal adhesion proteins in the embryo–maternal interaction (Klohonatz *et al.* 2019). Endometrial tissue from pregnant and non-pregnant mares was incubated in the presence of an embryo, inert beads, or peanut oil; the presence of beads did indeed trigger expression of focal adhesion molecules in the endometrium but did not suppress prostaglandin production. Meanwhile, co-culture of non-pregnant endometrium with an embryo resulted in both FAM expression and prostaglandin suppression, albeit at slightly different time-points (embryo days 9 and day 11, respectively). It is not yet clear if focal adhesion is an essential component of MRP or a concurrent event. It appears FAM expression alone is not enough to trigger prostaglandin suppression in the endometrium, with the caveat that this is a difficult process to mimic accurately under *in vitro* conditions or to examine closely *in vivo*.

Given the interactions between conceptus and endometrium observed *in vitro*, it seems unlikely that physical contact alone is responsible for the MRP in the horse but plausible that it does play a contributory role when considering evidence from other fields. An interaction has been discovered between laminar shear forces in an endometrial model and prostaglandin synthesis pathways, including an increased production of prostaglandin E2 in response to flow (Gnecco *et al.* 2019). In human endometrial organ-on-a-chip models, the endometrial response to progesterone (in terms of prolactin secretion and insulin-like growth factor binding protein-1 secretion) was potentiated under perfused (i.e. flow) vs static systems, showing that progesterone does not act alone in exerting its effects on endometrial function. Similar laminar shear forces could conceivably be experienced by mare endometrium due to conceptus migration, histotroph flow, and uterine contractility, although neither the amount of force required to elicit an endometrial response nor the specific mechanism underlying this process, has been documented.

Local vascular changes in response to embryo presence have been documented for the equine pregnancy. Silva* et al* used colour Doppler ultrasonography to map the vascular perfusion of the endometrium, which they found to be higher in pregnant mares (both horns, days 12–16) when compared to non-pregnant mares but also higher in the horn with an embryo present than the contralateral horn (days 12–15) (Silva *et al.* 2005). There are certainly documented mechanisms by which mechanical stimuli can trigger changes in vascularisation and tissue remodelling, both of which are crucial in the establishment of pregnancy. Clear overlaps exist between pathways activated by mechanical stress in tissues such as bone and those triggered by pregnancy in the equine endometrium. These include the up-regulation of growth factors, such as insulin-like growth factor-1 (IGF1) (Walters *et al.* 2001), vascular endothelial growth factor (VEGF) (Silva *et al.* 2011), transforming growth factor (TGF) β1 (Lennard *et al.* 1995), and bone morphogenetic proteins (BMP) 2 and 4 (Mikuni-Takagaki 1999, Hughes-Fulford 2004). Of note, these processes are known to be mediated via autocrine and paracrine mechanisms, often through the activation of tyrosine and serine/threonine kinase receptors; yet these systems have not been closely examined in the context of MRP and may provide a promising avenue for future investigations. Equine MRP research into these pathways has mostly focused on the level of gene expression (i.e. PCR and transcriptomics), but looking at post-translational modification of proteins (e.g. tyrosine and serine/threonine phosphorylation of kinases and receptors) could reveal an entire new cohort of interactions not evident at the gene expression (i.e. transcriptome) level. Determining these protein-level interactions could also unearth ways in which the embryo-endometrial dialogue could be mimicked or supported by pharmacological means and may provide new tools for monitoring the early pregnancy.

## Learning from other species: conserved pathways and new directions

While the mare has held on tightly to her MRP secret, the woman, cow, ewe and sow have gradually yielded theirs to prying scientists. Following on from those discoveries, the mechanisms by which MRP signals exert their effect on pregnancy maintenance have been examined in greater detail. Some of these mechanisms appear conserved across a wide range of mammalian species, and re-focusing from the ‘what’ to the ‘how’ of equine MRP could generate important information on early pregnancy in the horse. Studying the roles of conserved pathways also carries the advantage that many of the tools required to investigate them (e.g. inhibitors, antibodies) are readily available, along with pharmacological interventions that could prove useful in the reproductive context. Admittedly, it is hard to argue for a conserved mechanism across species given the striking diversity of known MRP molecules among well-studied species — from peptides to proteins to steroid hormones. Yet, the need to maintain luteal function for pregnancy is ubiquitous, thus at some point in the process, a range of non-conserved mechanisms must converge into a conserved pathway. For example, in both the mare and cow, luteal maintenance is preceded by a reduction in oxytocin receptor transcription in the endometrium and this facilitates the modulation of PGF_2α_ release (Hansen *et al.* 2017). Likewise, the MRP process in both species involves down-regulation of endometrial COX-2 (Xiao *et al.* 1999). Meanwhile, other steps in the process have been documented in the better-studied species but remain to be examined in the horse and could well harbour similarities that could provide important clues to the nature of the MRP signal. Numerous molecular actions have been attributed to IFN-τ in the establishment of pregnancy since its discovery; parallel phenomena have not yet been explored in the horse. These include, but are not limited to, the activation of the JAK/STAT pathway, involvement in the ERK1/2 cascade, and downstream expression of interferon-stimulated genes (ISGs).

Once IFN-τ was discovered in ruminants and confirmed to be the MRP signal, it took several years before the molecular pathways of its activity were clarified. It was known at the time, however, that type I interferons in other systems acted on the JAK/STAT signalling pathway. The JAK/STAT pathway has been widely studied and is relatively well characterised; it is involved in a plethora of developmental and homeostatic processes including those pertaining to ovarian function (Hall *et al.* 2018, Sutherland *et al.* 2018). Since interferons in this family all share the same receptor complex, it was considered likely that IFN-τ would participate in the same signalling pathways as the better-known IFN-α and IFN-β; thus JAK/STAT offered a clear avenue of investigation. Indeed, interferon receptor binding in the endometrium was found to engage the JAK/STAT pathway, driving the activation and repression of multiple interferon-stimulated genes with eventual down-regulation of COX-2 and finally a decrease in PGF_2α_ secretion (Binelli *et al.* 2001, Thatcher *et al.* 2001). Notably, JAK/STAT pathway involvement has been documented in human pregnancy, indicating it is not restricted to those species in which interferons mediate maternal recognition. Now considered a conserved mechanism across multiple species, STAT proteins play important roles in the establishment of pregnancy alongside maternal recognition/inhibition of luteolysis, for example, establishing uterine receptivity and regulation of maternal immune response (Maj and Chelmonska-Soyta 2007). The link between JAK/STAT signalling and the down-regulation of COX-2 expression/activity, with the latter being a key step leading to PGF_2α_ decrease in the mare, warrants investigation of this molecular pathway in the context of equine pregnancy. A thorough examination of the endometrial response to embryo secretions or physical conceptus-endometrial contact in the mare should determine whether the events leading up to COX-2 down-regulation are also driven through the JAK/STAT pathway.

JAK/STAT pathway activation happens through a series of post-translational modifications and protein binding rearrangements. In bovine endometrial epithelial cells, IFN-τ stimulated tyrosine phosphorylation of STAT proteins 1, 2 and 3; maximum phosphorylation was reached within 15 min and subsequently returned to control levels by 60 min (Binelli *et al.* 2001). This timeframe highlights the potential challenges associated with investigating transduction pathways mediated by phosphorylation and other post-translational modifications (PTMs) of proteins–such changes are easy to miss. In addition, PTMs are often reversible and unstable, with proteins rapidly becoming dephosphorylated during processing for analysis unless strict conditions are observed (e.g. temperature control, careful timing and prompt processing, use of appropriate phosphatase inhibitors). Whether through the JAK/STAT pathway or otherwise, equine MRP seems very likely to be driven by multiple steps of post-translational modification, rather than primarily transcription-level changes. This is well supported by the time scale on which MRP is known to occur —– evidenced by the rapid return to a non-pregnant state of endometrium when it is removed from immediate vicinity of the conceptus, and the need for constant migration of the conceptus to facilitate frequent direct interaction with all parts of the endometrial surface (as discussed above). New developments in mass spectrometry and emerging structural biology techniques will undoubtedly facilitate a comprehensive analysis of these processes.

More recently, new modes of IFN-τ activity are being revealed. Extracellularly regulated kinases (ERKs) are ubiquitously expressed across tissues and display conserved functions from bacteria to mammals, mediating cellular processes such as division, proliferation, apoptosis, and differentiation. Very direct evidence for involvement of the ERK1/2 signalling pathway in maternal recognition in sheep has been provided by infusion of an ERK1/2 inhibitor into the uterine lumen, where it blocked the action of IFN-τ (Lee *et al.* 2014). Prostaglandin pulses were restored and luteolysis progressed unimpeded, while oxytocin receptor expression was restored to non-pregnant levels. Because kinases and the associated signalling pathways, such as the ERK1/2 pathway, tend to be widely conserved and well studied, they present ready pharmacological targets. This provides scope for investigating these mechanisms in an *in vivo* setting, as was done in the sheep study. This also means that if indeed they are involved in the establishment of pregnancy, studying these pathways in the horse could have very practical implications as kinase-specific inhibitors are abundant and existing pharmaceuticals could be adapted to target reproductive performance of mares, support luteal function and perhaps reduce the incidence of early embryo loss.

Further to its anti-luteolytic actions, IFN-τ interacts with maternal endometrium by triggering expression of many ISGs that in turn promote uterine receptivity and conceptus implantation later on in pregnancy and stimulate conceptus elongation and IFN-τ production (reviewed by (Bazer *et al.* 2011)). Interestingly, expression of ISGs has been observed in non-ruminant species as well and has been proposed as a conserved mechanism that mediates protection of the conceptus from inflammatory insults across mammalian species (Hansen & Pru 2014). Whether this extends to the horse remains to be seen. Thus far, the study of ISGs in the horse has been limited to an initial investigation of ISG15, which showed that expression of ISG15 conjugated proteins in the endometrium did not differ between cyclic and pregnant mares 14 days after ovulation and day 50 of pregnancy (Klein *et al.* 2011). ISGs are numerous and varied, so a broader analysis of interferon-induced genes is required before conclusions about the role of ISGs in the equine pregnancy can be drawn.

The mechanisms discussed herein provide scope for targeted, hypothesis-driven investigation of potentially conserved aspects of pregnancy maintenance or failure in the horse and could prove a fruitful avenue for future research, regardless of whether the MRP signal itself is identified. In light of their extensive documentation in other species or processes and some cases propensity for pharmacological intervention, these and other mechanisms could very well reveal more about the biology of pregnancy in this species than the discovery of a single molecule/factor responsible for maternal recognition.

## Embracing complex answers: embryo–-maternal dialogue in the ‘omics era

Within the equine research community, some have proposed that the MRP has not been discovered because it simply does not exist. Others have suggested that the equine MRP is not one single chemical compound but an interaction of multiple processes, contrasting this with the seemingly straightforward mechanisms employed by ruminants, pigs, and humans. Reproductive biology textbooks present what seems a direct and linear relationship between MRP signals in these species (IFN-τ, oestradiol, and hCG, respectively) and the protection of the corpus luteum from luteolysis. Yet, upon closer inspection, MRP in all well-studied species is emerging as a more sophisticated process and is embedded in an intricate network of events that orchestrate early pregnancy. IFN-τ in ruminants does not act directly on the CL but employs multiple signalling pathways, supports pregnancy through both paracrine and endocrine routes, and even participates in a two-way dialogue whereby maternal factors further enhance conceptus IFN production (reviewed in (Roberts *et al.* 2008, Hansen *et al.* 2017). In a transcriptome study of the effects of IFN-τ on ovine endometrial cells, 356 genes were up-regulated and 229 genes were down-regulated by IFN-τ treatment, highlighting the complexity of the effects (Chen *et al.* 2007). Affected genes included those involved in prostaglandin metabolism, growth factor production, apoptosis, extracellular matrix remodelling, angiogenesis, blood coagulation and inflammation. Clearly, a linear MRP process is not the only role for IFN-τ in the physiology of early pregnancy. Likewise, hCG has been implicated in an array of critical pregnancy support functions beyond luteal survival including embryo–uterine dialogue, immune tolerance and priming of the endometrium for implantation (reviewed in (Fournier *et al.* 2015).

Perhaps the difference between the horse and the other domestic species lies not in the innate biological complexity but in the investigational methods and approaches used historically in the different species. In the case of the horse, maybe we have missed the window of time when scientific research was done in a way that was ‘simple’ enough for us to accept straightforward evidence for a straightforward concept. Whilst the scientific method remains fundamentally the same, the tools we use have changed dramatically in the last ~50 years and the type and quantity of data we generate are strikingly different. Examples of this include the vast datasets generated using emerging genomic, transcriptomic and proteomic platforms and high throughput approaches. ‘Shotgun’ or ‘discovery’ approaches have become more commonplace in contrast to hypothesis-driven, targeted studies of single hormones, proteins or pathways that formed the majority of studies in the latter half of the 20th century. ‘Omics’ is a term that has evolved to collectively refer to technologies capable of examining *en masse* the entire profile of genes, transcripts, proteins, lipids or metabolites representative of a cell, fluid or physiological state —– despite the occasional erroneous reference to small scale target studies of, for example, proteins or lipids as ‘proteomic’, or ‘lipidomic’ analyses.

Immunoblots and immunoassays, as means to detect and quantify expression of a single protein in focus, are being overtaken by mass spectrometry-based proteomic analysis of thousands of proteins in every sample or treatment group. Laborious PCR analysis of a handful of individual genes is often replaced by microarray approaches or, more recently, large-scale NextGen sequencing. Thousands of proteins, transcripts and non-coding RNAs are up- or down-regulated in response to *in vitro* experimental treatments or between *in vivo* physiological states. The vast amounts of data these technologies generate would have been inconceivable a mere 30 years ago and certainly were not employed in the early studies of MRP in domestic species and humans.

In the horse, gene expression (de Ruijter-Villani *et al.* 2015, Klohonatz *et al.* 2015) and transcriptome (Klein *et al.* 2010, Klein & Troedsson 2011) studies have been instrumental in characterizing the early embryo, receptive endometrium and their interactions; now, the advent of mass spectrometry technologies and the sequencing of the equine genome (Wade *et al.* 2009) have opened new doors for investigating the fundamental workhorses of biological interactions – the proteins themselves. Proteomics presents a promising avenue for studying embryo–maternal interactions as proteins are responsible for enzymatic activity, receptor-driven interactions and signalling cascades, all of which respond rapidly to cues within the immediate physiological environment. Recent improvements in the sensitivity, mass accuracy and resolution of mass spectrometers (Scigelova & Makarov 2009) have meant that ‘bottom-up’ shotgun proteomics workflows can be applied directly to miniscule samples, whose total protein content would previously have been considered insufficient for analysis.

Earlier attempts to examine the equine embryo secretome had been limited to small numbers of proteins of focus (Aggarwal *et al.* 1980, Herrler *et al.* 2000, Albihn *et al.* 2003, Bemis *et al.* 2012); on one occasion, culture media were collected from 10, 12, 14 and 16 day embryos for proteomic analysis but no protein was detected in 10 day culture media using the chosen methodology (SDS-PAGE and silver staining), while other samples yielded a small number of proteins but (apart from uterocalin) these were not assigned protein or gene IDs and thus remained unidentified (Budik *et al.* 2012). Our study of the proteomics of equine pregnancy was the first successful shotgun proteomics analysis of the proteins released by the early (day 8-10) equine embryo into its immediate environment, whereby we were able to detect 72 (24 h culture) and 97 (48 h culture) unique protein IDs in the embryo secretome, 732 protein IDs in blastocoel fluid, and 11 proteins IDs in the embryo capsule (Swegen *et al.* 2017). Among these were a pregnancy-specific proteinase (PAG) secreted by embryos at day 10, along with a prostaglandin receptor inhibiting protein (PTGFRN) and a progesterone co-factor factor (FKBP4) detected in blastocoel fluid.

A similar proteomics approach was used to analyse the proteins within the embryonic yolk sac and uterine luminal fluid of pregnant mares on day 13 (Smits *et al.* 2018). Among 1153 proteins identified, 119 proteins were differentially expressed in the uterine fluid of pregnant mares compared to cyclic mares. Excitingly, both the embryo secretome and uterine fluid studies identified multiple proteins capable of inhibiting prostaglandin synthesis/function and those that assist progesterone in its receptor interactions. The next challenge will be to define which of these proteins functionally interact with the maternal endometrium in a way that aligns with existing knowledge about the equine MRP.

Whilst we are unlikely to have covered the complete, complex mixture of proteins in these fluids and embryo capsule, these profiles should nonetheless serve as a useful resource for future research initiatives and may be valuable in comparative multispecies analyses seeking to understand evolutionary aspects of reproductive function. Many of the proteins identified have putative or reported roles in pregnancy or related processes and could serve as a nidus for countless new ideas, hypotheses and directions of scientific investigation. Meanwhile, integration and co-analysis of these proteomic results with the transcriptomic analyses of the pregnant endometrium (Gebhardt *et al.* 2012, Klein *et al.* 2010, Klein & Troedsson 2011, Klein 2015 *b*, Smits *et al.* 2020) promise to yield valuable new insights by highlighting specific networks and pathways that appear to become more active in the pregnant state.

Transcriptomics and proteomics will continue to evolve and generate data of greater depth. We are also likely to see an expansion of the equine reproduction ‘omics repertoire; characterisation of non-coding RNAs, lipidomic analyses and the next rung of proteomics—–the phosphoproteome along with other post-translational modifications—–are likely to be next in line. In the realm of human fertility, NMR-based and mass spec-based lipidomics approaches have already been flagged as promising tools to ascertain endometrial receptivity (Vilella *et al.* 2013). Lipidomics in equine reproduction has also surfaced; Wood* et al* have begun lipidomic analysis of mare amniotic fluid (Wood *et al.* 2018). In early equine pregnancy, the central role of prostaglandins, which belong to the eicosanoid subgroup of lipids, makes lipidomics an inviting direction for further documenting embryo-maternal interactions. Phospholipids such as platelet-activation factor (PAF) have been identified very early in the post-fertilisation phase in many species and appear to have essential roles in early pregnancy (Ryan *et al.* 1992). Comprehensive lipidomic analysis of early equine pregnancy is bound to reveal new dynamics in the secretion and interactions of these vital bioactive molecules.

An emerging and as yet underappreciated value of ‘omics data is the opportunity to revisit reposited datasets from previous studies and analyse them in new ways to glean new information. Some datasets may become profoundly more useful with time and once other studies, for example in other species, tissues or time-points, become published and allow interesting comparative analyses that combine data from different projects. This was elegantly demonstrated in the study of equine pregnancy when a series of bioinformatics techniques were deployed to re-examine previously published endometrial transcriptome data in the early pregnancy of cattle, pigs and horses (Bauersachs & Wolf 2012). Overlapping genes pointed to conserved mechanisms in early pregnancy but unique features and some surprising findings were revealed for each species. Most recently, Smits* et al* integrated comprehensive novel miRNA sequencing data of pregnant and control mare endometrium, uterine fluid, embryonic tissue and yolk sac fluid with corresponding transcriptome (mRNA) and proteome datasets (Smits *et al.* 2020) on day 13 post-ovulation. This approach was particularly interesting as it encompassed multiple tissues/fluids, and three levels of the transcription/translation cascade, yielding important information on the origins and regulation of protein expression. For example, comparison of the pregnancy-induced proteins in the histotroph with differentially expressed genes between the embryo and the endometrium indicated embryonic origin of 70% of the proteins in the uterine fluid. Such integrative and comparative studies will become even more informative as we fill in the gaps of genome annotation and quantitative RNA sequencing technologies are more widely and more uniformly applied across different species. Bioinformatic approaches will be extremely useful not only in studying conserved mechanisms of pregnancy recognition across species but in directing researchers towards the physiological pathways that deserve more targeted study.

This is an exciting new era in science; although we are some years away from ‘omics technologies reaching their peak potential, we can expect that depth and sensitivity will continue to improve and, perhaps most importantly, improved tools to interpret these vast datasets will become available. Currently, the lack of such interpretation tools presents the greatest limitation. Even proteomics relies on gene ontology for meaningful analysis,that is, what is already known about the structure and function of each gene/protein in commonly studied species, while equivalent tools for lipidomics, miRNA transcriptomics, and phosphoproteomics analyses, for example, are yet to become readily available and user-friendly. Ultimately, the advent of ‘omics means that we can no longer expect a single response to a single treatment or biological event. The data these fields generate are increasingly complex, often frustratingly so. We must not shy away from this but understand that these datasets are far more reflective of the complexity of biology than previous scientific approaches were. We must learn to manage, interpret and embrace these results; a shift towards thinking in terms of networks and dynamic systems rather than linear pathways and binary on/off switches should be encouraged.

## Enlisting new tools: *in vitro* strategies for endometrial studies

Of course, nothing can replace a real horse (*in vivo* study) and unquestionably, the early studies using whole animals were indispensable to our understanding of pregnancy—–in particular, of endocrinological relationships, which had been the focus of much research in the 20th century. However, disentangling the direct effects of the conceptus from the systemic effects of endocrine events (i.e. effects downstream of the CL/progesterone) is particularly challenging in a whole animal system. Thus to truly decipher the processes arising directly from embryo-maternal interaction, *in vitro* models are essential. Traditional cell culture relies largely on monocultures consisting of a single cell type in a single layer. However, *in situ* endometrium comprises luminal and glandular epithelial cells intricately arranged into glands responsible for the secretion of numerous substances (including PGF_2α_, and histotroph in early pregnancy) with an underlayer of stromal cells. The three-dimensional architecture of this tissue, its secretory activity, cell polarity and the interactions between its constituent cell types are integral to its function, meaning traditional cell culture systems involving single cell type monolayers are of limited use for studying the interaction between embryo and endometrium, even where a co-culture with early embryos or conceptus membranes can be supported. Nevertheless, experiments based on this type of cell culture were fundamental in establishing the endometrial epithelium as primarily responsible for producing PGF_2α_ along with the direct effects of oxytocin on both epithelial and stromal cells of the equine endometrium (Watson *et al.* 1992).

Culturing tissue explants, where a small piece of endometrium would be used without isolating individual cell types, was expected to overcome some of the limitations of monocultures by maintaining three-dimensional tissue architecture and thus, secretory activity of the endometrial glands. Indeed, several key studies establishing the role of PGF_2α_ secretion and its modulation by the presence of conceptus tissues were performed using such explants (Vernon *et al.* 1981, Berglund *et al.* 1982, Sissener *et al.* 1996). However, explants suffer from the distinct disadvantage of requiring a primary source of tissue and short lifespan. Furthermore, while secretory activity generally signalled viable tissue, cell viability and normal function were rarely confirmed in studies relying on this experimental strategy. Somewhat alarming was the finding that explants start to show necrotic change from as early as 12 h of culture (Schwinghamer *et al.* 2018), even though it was not uncommon for studies to use time-points of 24 h and later in explant culture experiments. Even more concerning was the marked increase in PTGS2 expression concurrent with degenerative changes in the endometrial tissue in culture, presenting a significant confounding issue (and major obstacle) to the study of prostaglandin synthesis and secretion with the aid of an explant model.

In short, both techniques—–monolayers and tissue explants—–suffer from a short useful lifespan and inadequate functional resemblance of *in vivo* endometrium. Fortunately, innovative *in vitro* modelling strategies are coming to the rescue. Endometrial epithelium harbours a range of cell differentiation states, including a reserve of progenitor cells—stem-like cells with a defined cell fate but still capable of proliferation. The precise identity of these cells is still under investigation (Nguyen *et al.* 2017); however, they have already been successfully exploited to generate human endometrial epithelial organoids (Turco *et al.* 2017). Organoids have been dubbed ‘miniaturised organs’ and consist of a three-dimensional extracellular matrix scaffold populated by cells that are capable of self-organisation into complex structures closely mimicking the tissue of origin; such models have been established for a range of tissues including the brain, kidney, intestine and liver. The technique represents a vast improvement on the previously available strategies. Excitingly, not only do human endometrial organoids organise into glandular structures with appropriate cell polarity, these glands secrete a glycogen-rich substance reminiscent of histotroph. The structures have also been thoroughly validated to respond to the different hormonal environments experienced by endometrium throughout the menstrual cycle in a manner synchronous with *in vivo* endometrium, as determined by extensive RNA profiling. Of particular significance to those interested in MRP is the observation that endometrial organoids respond to treatment with hCG and shift to a pregnant endometrium phenotype (Turco *et al.* 2017). Excitingly, such an* in vitro* model has now also been validated for the horse (Thompson *et al.* 2020), although how equine endometrial organoids interact with the embryo and its secretions is yet to be examined. Organoids may even help to clarify the mechanical interaction between embryo and endometrium: human endometrial organoids have been confirmed to functionally express the mechanosensitive PIEZO1 channel, mimicking the ion channel profile and mechanoreceptor signalling of in situ endometrium (Hennes *et al.* 2019). Organoid cultures represent a new era of *in vitro* modelling and herald fresh hope for equine embryo-maternal interaction research.

More recently, the same group that pioneered endometrial organoids validated another strategy to enhance the study of embryo–maternal interactions: trophoblast organoids (Turco *et al.* 2018). These organoids form villous-like structures, secrete placenta-specific peptides and hormones including hCG, and their methylation patterns closely resemble those of a normal first-trimester placenta. Such a system should be invaluable for investigating trophoblast interactions with the maternal environment. An equivalent strategy as it relates to the mare is yet to be established but would be extremely useful in answering many questions about embryo-maternal interactions, early placentation and pregnancy-compromising pathologies.

Elsewhere researchers have turned to strategies such as 3D printing and microfluidics to generate organ-on-a-chip models for reproductive tissues including bovine oviduct (Ferraz *et al.* 2018) and human endometrium (Gnecco *et al.* 2019). Further development of these approaches and application in the horse will be invaluable for understanding the effects of subtle and dynamic changes in the uterine microenvironment, particularly where pulsatile release of hormones and/or shear forces play a physiological role and prove particularly challenging to replicate in traditional *in vitro* systems.

Following a phase of thorough validation and undoubtedly the overcoming of a handful of species-specific challenges, three-dimensional endometrial culture strategies promise to be transformative for our research not only of early pregnancy but also of endometrial pathology and of this tissue’s responses to sperm, seminal plasma, semen extenders, pathogens, and inflammatory mediators.

## Adjusting expectations: MRP as academic pursuit vs clinical panacea

The mystery surrounding equine pregnancy has both frustrated and fascinated researchers and clinicians alike. The term ‘maternal recognition’ refers specifically to recognition by the maternal system and not to the *detection* of pregnancy. The unknown MRP signal is often presented as a ‘holy grail’, which, once discovered, will provide answers to the practical problems around early pregnancy testing and high rates of early embryo loss facing the horse breeding industries. Yet such issues persist in other species, despite a much more thorough understanding of their MRP mechanisms. Cattle continue to suffer high rates of early pregnancy loss, and IFN-τ has not transpired into a means for early pregnancy testing in ruminants. In women, pre-implantation embryo loss is estimated at 10–40% and overall pregnancy loss from fertilisation to birth is approximately 40–60% (Jarvis 2016), despite hCG having been discovered several decades ago. What, then, is a realistic expectation for how better understanding the equine MRP might benefit clinical practice?

Human chorionic gonadotrophin was discovered when Aschheim and Zondek in the 1920s found that the urine of pregnant women could induce oestrus when injected into an immature rat or mouse, and demonstrated the ability of this novel substance to prolong the luteal phase in women. It was not until the 1980s that the same substance was confirmed to be secreted by the human embryo and could thus be deemed the MRP signal of the human species (Ross 1979, Fishel *et al.* 1984). hCG is produced by the cytotrophoblast cells, which invade the maternal endometrium upon implantation of the blastocyst, and later contribute to the formation of the placenta. Thus, hCG becomes detectable in the systemic circulation as soon as implantation occurs 8–10 days after ovulation. From here onwards, hCG concentration in the urine doubles every two to three days during early pregnancy. Its abundance and ready detection in the urine and blood of pregnant women made hCG very useful for pregnancy diagnosis, well before its role in MRP was fully documented.

We must note here that placentation in humans differs drastically from that of equids, which do not experience the same level of invasion of maternal tissue by the conceptus. Some degree of interdigitation does take place but this occurs later in the pregnancy (around day 40); thus it is clear that any chemical signal released by the conceptus would be subject to a distribution pattern very different from that of hCG in the early human pregnancy. The equine embryo remains suspended, mobile and completely devoid of any physical anchoring mechanism or vascular invasion in these early stages. Thus any compound involved in signalling during MRP, even if secreted in relatively high abundance by the conceptus, is unlikely to be detectable in the maternal circulation as it would only be able to get there by diffusion through the endometrium and would rapidly become diluted by the large circulating volume. Evidence gathered thus far would suggest that this compound acts directly on the endometrium to trigger changes in prostaglandin release, so its inability to enter the circulation is biologically inconsequential but of course hinders our capacity to detect and identify it, and limits the MRP signal’s potential usefulness as clinical biomarker of pregnancy. This has very much been the case in ruminants, where INF-τ acts within the uterine environment but is barely detectable in extrauterine tissues, meaning it has not been useful for pregnancy detection. Instead, downstream of IFN-τ, IFN-stimulated genes (ISGs) are being explored as targets for a pregnancy test but so far have not yielded clinically adequate results (Mauffre *et al.* 2016). Such an approach would not be particularly helpful in the horse as the timing of MRP and downstream events would not provide any advantage over routinely used ultrasound exams carried out at day 12–14 post-ovulation, unless of course, it came with extra benefits such as detection of foetal sex.

The MRP signalling molecule itself is thus very unlikely to serve as a pregnancy test target in the horse. This is not to say that a pregnancy test is not possible. Embryo–maternal communication in the horse begins at the point of a peculiar process whereby only fertilised ova are permitted to descend through the oviduct into the uterine lumen on day 6 (Betteridge & Mitchell 1972, 1974, Betteridge *et al.* 1979) and one can speculate that subtle systemic changes could occur as a result of this interaction, even if we have yet to detect them. Even as early as day 3, the serum of pregnant mares acquires biochemical characteristics distinct from those of non-pregnant mares; this is illustrated by the ability of pregnant mares’ serum to inhibit erythrocyte-lymphocyte rosette formation via the rosette inhibition assay (Ohnuma *et al.* 2000). The phenomenon has been attributed to an elusive protein or ‘early pregnancy factor’ and we must note here that while a pregnancy test based on the yet-unidentified ‘factor’ proved unsuccessful, the findings relating to rosette inhibition remain relevant albeit the factor(s) responsible for the altered chemistry still unknown. Thus, a yet undefined but seemingly consistent systemic change takes place in pregnant mares much earlier than the MRP; in the context of pregnancy diagnosis, investigating these earlier changes may hold more clinical value than the MRP at 10–14 days post-ovulation.

The more we know about embryo–maternal interaction and early pregnancy in general, the more clinical use we are likely to glean, whether through the MRP signal itself or co-occurring aspects of embryo-maternal interaction. The latter may prove more useful; as an example, shotgun proteomic analysis of early pregnancy uterine luminal fluid revealed significant enrichment at the time of MRP of the chaperone protein FKBP4 (Lawson *et al.* 2018). This protein co-operates with HSP90 to form functional steroid receptor complexes and is essential for activation of the progesterone receptor by progesterone (Tranguch *et al.* 2006). An essential co-factor for progesterone action, its absence in mice causes complete infertility by way of uterine progesterone insensitivity, lack of uterine receptivity and consequent failure of implantation (Tranguch *et al.* 2005). There is also an association between human early pregnancy loss and FKBP4 expression deficit (Chen *et al.* 2015). Progestin supplementation of mares suffering a history of early embryo loss is commonplace in equine breeding practice, but co-factors such as FKBP4 and HSP90 have not been examined as therapeutic targets for fertility intervention —– even though they are known to form a critical, previously overlooked mechanism in progesterone function. Furthermore, as controversy surrounds the relationship between systemic progesterone levels, progestin administration and pregnancy outcome in the mare, perhaps the investigation of progesterone function (e.g. aberrations in chaperone function/expression), rather than quantity, could provide a more nuanced and fruitful approach for future research into the role of progesterone in early pregnancy failure.

To take FKBP4 as an example, this co-factor has been hailed as a promising therapeutic target (in mouse and human studies) due to the specificity of its interaction with steroid hormone receptors, opening avenues for both contraceptive and fertility-enhancement strategies (Sivils *et al.* 2011, Guy *et al.* 2015). In our study of equine pregnancy proteomics, enrichment of FKBP4 in the uterine lumen at day 14 of gestation vs later pregnancy time-points suggested that progesterone-potentiating mechanisms could be at play in the mare around the time of maternal recognition. It is not yet clear whether FKBP4 is actively secreted into the luminal fluid, or is incidentally released from the endometrial epithelium. We also detected FKBP4 in the blastocoel fluid of 10 day old equine embryos (Swegen *et al.* 2017), and corresponding RNA transcripts have previously been documented in the 16 day old equine conceptus (Klein 2015 *b*); this suggests that FKBP4 originates within the conceptus and raises the intriguing possibility that the embryo itself assists progesterone action in the early pregnant uterus, with novel implications for an embryo–maternal signalling role of FKBP4. Further studies would determine the functional significance of this critical progesterone receptor co-chaperone in equine pregnancy, potentially revealing useful attributes for reproductive management and in turn allowing the refinement of current empirical treatments for pregnancy support in the mare.

Extrapolating on what we know about MRP in other species, understanding the equine MRP may have other clinical benefits, even if it is unlikely to yield an early pregnancy test. One rather curious observation is that the production of IFN-τ differs between male and female bovine conceptuses at the same stage of development (Larson *et al.* 2001). The difference is evident when comparing embryos grouped according to sex, but overall too variable among individuals to serve as a standalone assay capable of determining the sex of a given embryo. The sexually dimorphic production of IFN-τ appears to be intricately linked with metabolic function genes located on the X-chromosome, which undergoes X-inactivation in females at the late blastocyst stage (Kimura *et al.* 2004). It will be fascinating to see whether a similar relationship exists within the mechanism of equine maternal recognition. At a population level, there have been multiple reports of the maternal nutritional milieu influencing sex ratios of offspring in horses; mares in better body condition or subjected to an increasing plane of nutrition are more likely to produce colts (Monard *et al.* 1997, Cameron *et al.* 1999, Cameron & Linklater 2007). The mechanism is currently unknown although maternal glucose levels at conception and early pregnancy have been tentatively implicated in the process, with early equine embryos displaying sexually dimorphic expression of insulin-like growth factor-1 (Beckelmann *et al.* 2013). The evidence from both cattle and horses suggests there are biological differences between male and female embryos that justify an interest in the embryo secretome beyond seeking to identify the MRP; understanding how embryos respond to their immediate environment will lead to enhancements of *in vitro* production conditions (e.g. customised media to support development of male or female embryos), tools to non-invasively assess embryo quality and possibly those to determine embryo sex at the time of embryo transfer.

Another potential clinical use of embryo-secreted factors, including but not restricted to the putative MRP signal, lies in non-invasive embryo health monitoring. Noting here that embryo health is a continuum rather than a binary pregnant/non-pregnant outcome, it is becoming increasingly apparent that a range of maternal and, indeed, paternal factors can affect the health of offspring, including diet, ageing, exposure to environmental pollutants, *in vitro* gamete manipulation and many others, collectively termed ‘developmental origins of health and disease’ or DOHaD (Fleming *et al.* 2015, Duranthon & Chavatte-Palmer 2018). It is also now emerging that many of these impacts occur remarkably early in development, and some are mediated through effects on the contributing gametes, such as the consequences of ageing and oxidative stress on spermatozoa and oocytes culminating in metabolic aberrations, altered epigenetic status and other changes in early embryos (Jenkins *et al.* 2015, Woods *et al.* 2018, Carnevale *et al.* 2020, Yoshizaki *et al.* 2021). In this context, monitoring embryo health is set to become an important tool both in studying and diagnosis of DOHaD related conditions, particularly in light of the horse’s role as an elite athlete, wherein even minor developmental defects can compromise performance potential. In ruminant species, an embryo’s capacity to release of IFN-τ has been suggested as a possible indicator of embryo health during *in vitro* culture; however, reports have been conflicting as to whether increased secretion is a marker of good health, delayed development, or a result of metabolic or oxidative stress. Meanwhile, analysis of human embryo-conditioned media identified apolipoprotein A1 (APOA1) as the major protein quantitatively correlated with pregnancy outcome and thus a possible biomarker of embryo developmental potential (Nyalwidhe *et al.* 2013). Lower levels of secreted APOA1 were consistently associated with a higher likelihood of viable pregnancy, suggesting that an embryo’s capacity to bind and/or internalize APOA1 might be reflective of its metabolic competence. In our study of equine embryo-secreted proteins, APOA1 was one of the dominant proteins detected in embryo-conditioned media, along with several other apolipoproteins and low density lipoprotein-related proteins in embryo-conditioned media and blastocoel fluid, and an apolipoprotein binding receptor, ABCA1, in the embryo capsule (Swegen *et al.* 2017). Further profiling of equine embryo-secreted apolipoproteins (including their lipidation status and microRNA cargo) in healthy vs metabolically ‘stressed’ embryos will reveal if a similar potential for non-invasive embryo quality monitoring exists in the horse. As detection techniques become more refined and more sensitive, we should be able to develop screening protocols for *in vitro* embryo production and embryo transfer programs. There is no indication, however, that the MRP signal is any more likely than any other secreted factor to serve as such a biomarker, thus the research emphasis here must be on the functional correlation with embryo competence, DOHaD factors/embryo health and pregnancy outcome rather than, specifically, a role in maternal recognition.

## Concluding remarks

We have come a long way towards understanding the mechanism of maternal recognition in the mare. Importantly, we are beginning to acknowledge that this diverse process in all species is more resemblant of a web rather than a simple linear chain of events and I hope we can continue to embrace the complexity of early pregnancy without abandoning the scientific narrative that makes it all comprehensible and fascinating. Maternal recognition in the horse undoubtedly exists, likely involves a combination of chemical and mechanical signalling and probably consists of a relatively unstable, dynamic interaction of multiple pathways. Many excellent and relevant tools have emerged in disparate fields of science that promise to infuse new vigour into the quest for the equine MRP. A reliable and consistent *in vitro* model that mimics the anti-luteolytic effects induced by the early embryo will be crucial and seems achievable given the recent advances in studies of the human endometrium together with the robust evidence for inhibition of prostaglandin synthesis as a keystone intermediary of MRP in the mare. Alas, a word of caution to those who expect a radical transformation of the clinical landscape of the equine pregnancy once an MRP signal is pinned down: historically this has not been the case in other species, and there is little evidence to suggest that discovery of equine MRP mechanisms will directly reduce early pregnancy losses. Re-framing the quest to focus on embryo-maternal interactions as a whole, along with other essential early pregnancy functions, is bound to be more fruitful in terms of translational outcomes. Finding the enigmatic MRP signal of the horse remains a worthwhile pursuit, with the caveat that we must remain open to far more useful and interesting discoveries about early pregnancy along the way. Let the search continue and be full of wonderful surprises.

## Declaration of interest

The author declares that there is no conflict of interest that could be perceived as prejudicing the impartiality of the research reported.

## Funding

This work did not receive any specific grant from any funding agency in the public, commercial or not-for-profit sector.
